# Successful treatment of severe accidental hypothermia with cardiac arrest for a long time using cardiopulmonary bypass - report of a case

**DOI:** 10.1186/1865-1380-5-9

**Published:** 2012-02-02

**Authors:** Keigo Sawamoto, Katsutoshi Tanno, Yoshihiro Takeyama, Yasufumi Asai

**Affiliations:** 1 Traumatology and Critical Care Medicine, Sapporo Medical University, Sapporo, Japan

## Abstract

Accidental hypothermia is defined as an unintentional decrease in body temperature to below 35°C, and cases in which temperatures drop below 28°C are considered severe and have a high mortality rate. This study presents the case of a 57-year-old man discovered drifting at sea who was admitted to our hospital suffering from cardiac arrest. Upon admittance, an electrocardiogram indicated asystole, and the patient's temperature was 22°C. Thirty minutes of standard CPR and external rewarming were ineffective in raising his temperature. However, although he had been in cardiac arrest for nearly 2 h, it was decided to continue resuscitation, and a cardiopulmonary bypass (CPB) was initiated. CPB was successful in gradually rewarming the patient and restoring spontaneous circulation. After approximately 1 month of rehabilitation, the patient was subsequently discharged, displaying no neurological deficits. The successful recovery in this case suggests that CPB can be considered a useful way to treat severe hypothermia, particularly in those suffering from cardiac arrest.

## Introduction

In the clinical setting, it is often difficult to determine whether hypoxia associated with submersion or severe accidental hypothermia associated with immersion is the cause of cardiac arrest due to drowning. We here report the case of a patient who developed prolonged cardiac arrest because of drowning in the sea, a situation in which one is stumped concerning resuscitation. Using cardiopulmonary bypass (CPB), resuscitation was achieved, and the patient had no neurological deficits.

## Case report

**Figure 1:**
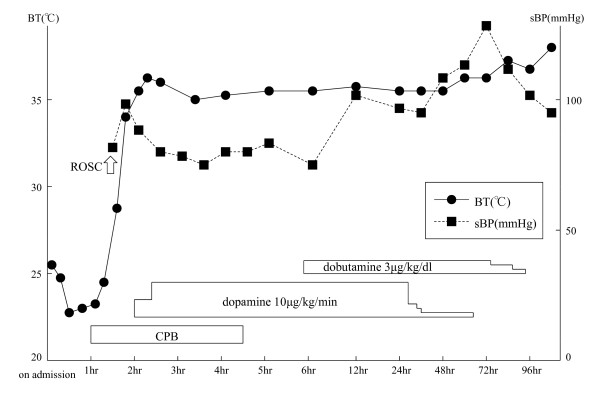
**Clinical course**.

**Figure 2:**
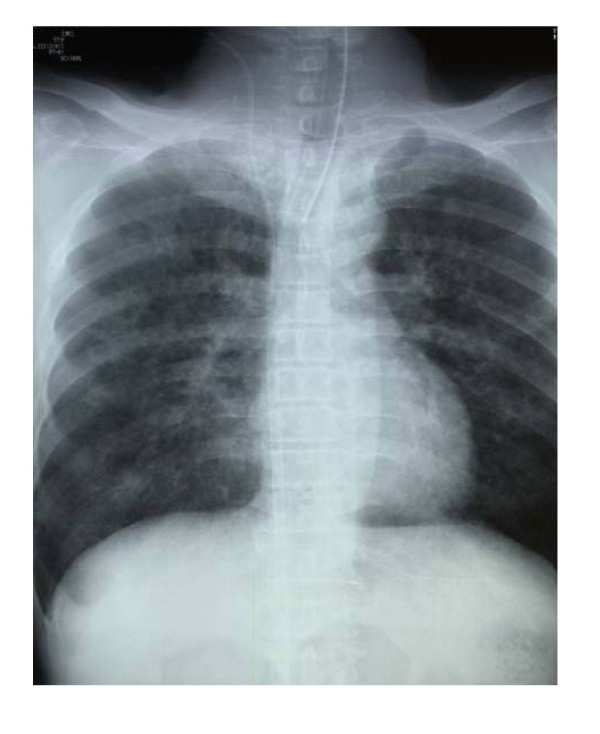
**Chest X-ray on admission**.

**Figure 3:**
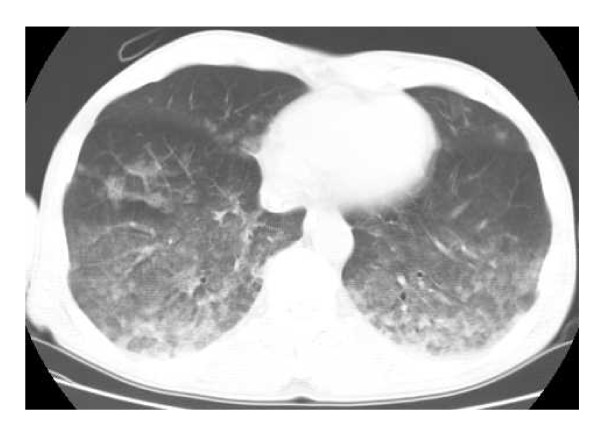
**Chest computed tomography on admission**.

**Figure 4:**
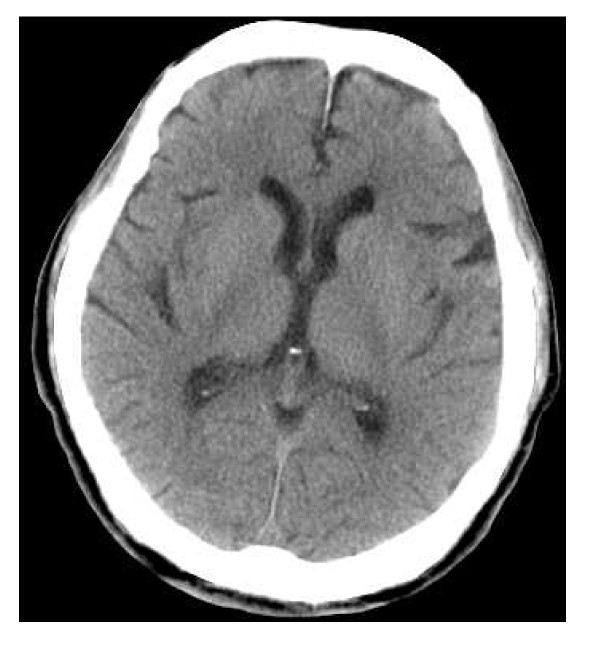
**Head computed tomography on admission**.

In June 2008, a 57-year-old male was found drifting in the sea at 08:07 a.m. The seawater temperature was 12°C. Emergency medical technicians confirmed his cardiac arrest at the port at 08:28 a.m., and his electrocardiogram showed asystole. He was brought to our emergency department (ED) at 08:51 a.m. A core body temperature of 22.0°C was registered in the rectum, and his pupils were fixed and dilated (Figure 1). Although we continued standard CPR with tracheal intubation and external rewarming using warmed infusions and radiant heat, the patient's temperature remained at 22.8°C 30 min after arrival. In addition, sputum comprising massive bubbles resembling seawater was evident in his endotracheal tube. Because he had been in cardiac arrest for at least 90 min, we were stumped about whether to continue resuscitation or not at that time. However, we found that spontaneous slight gasping breathing without a pulse and chest compressions appeared at 09:24 a.m. We then decided to apply CPB (cannulated from right femoral vessels) for rewarming and circulation because we suspected that the cause of his cardiac arrest was severe accidental hypothermia rather than hypoxia due to drowning. After we started CPB at 09:55 a.m., although his electrocardiogram showed asystole at first, it changed to ventricular fibrillation (VF) of low amplitude as his temperature rose, and its amplitude slowly increased. His condition changed from VF into sinus rhythm without defibrillation at the time point when his temperature reached 26.7°C (10:22 a.m.). Soon, movement of his limbs appeared, the size and reactivity of the pupils became almost normal, and spontaneous breathing became adequate. Aspiration of a large amount of seawater was suspected from the thoracic radiography (Figures 2, 3). However, head CT showed no hypoxic changes such as diffuse swelling at that time (Figure 4). CPB was discontinued at 01:25 p.m. because of his hemodynamic stability with catecholamine treatment, which was started at 34°C. After neurological rehabilitation, he was discharged without any neurological deficits on day 32.

## Discussion

Accidental hypothermia is defined as an unintentional decrease in body temperature below 35°C [1]. Severe accidental hypothermia (core temperature below 28°C) is still associated with a high mortality rate ranging from 30-80% [2,3]. Major causes of severe accidental hypothermia are drowning (submersion or immersion), being caught in an avalanche, and exposure to cold air. Submersion is associated with hypoxia because of sinking until being completely covered with water. Immersion is associated with accidental hypothermia because of sinking until being covered with water except for the face. As has been well described, since 'a hypothermic patient is not dead until warm and dead,' resuscitation should be continued in the hospital until the patient has been rewarmed to 33-35°C [3-5]. It is common knowledge that low temperature increases the ischemic tolerance of the brain. Several authors have described remarkable neurologically intact recovery after prolonged cold-water immersion [3,4,6]. However if asphyxiation precedes cardiac arrest, even in the hypothermic patient, the chances of survival seem to be less, because hypothermia cannot render its cerebral protective effect [1-6]. Therefore, in such cases, active treatment cannot have a sufficiently positive outcome.

There is no consensus on reliable prognostic indicators to determine the efficacy of active rewarming for hypothermic cardiac arrest patients [4]. Therefore, most emergency physicians continue efforts to resuscitate for some time, as previously indicated. Recently, it has been reported that active internal rewarming using CPB is useful for resuscitation in cases where severe accidental hypothermia develops into cardiac arrest [7]. However, it is generally difficult for all emergency departments to use CPB because of limitations in the availability of CPB or lack of manpower. It can be speculated that in many cases resuscitation is discontinued without waiting for rewarming to occur.

Several investigators have reported on prognostic factors likely to identify patients in hypothermic cardiac arrest who would probably benefit from resuscitation by CPB, although these have been small retrospective studies [2,5,7-9]. Farstad et al. analyzed 26 hypothermic cardiac arrest patients resuscitated by CPB and suggested that extreme hyperkalemia (serum potassium > 10 mmol/l) as a sign of cellular damage indicates a dismal prognosis [2]. Mair et al. analyzed 22 hypothermic cardiac arrest patients resuscitated by CPB and suggested that plasma potassium levels (serum potassium > 9 mmol/l), central venous pH (pH < 6.50) and ACT (activated clotting time > 400 s) on admission can be used to identify hypothermic arrest victims in whom death preceded cooling [5]. Hauty et al. analyzed ten severely hypothermic patients rescued from a snow-covered mountain and resuscitated by CPB, and concluded that hyperkalemia (> 10 mmol/l) and markedly elevated serum ammonia levels (> 250 mcmol/l) predict a dire outcome [8]. Silfvast et al. analyzed 23 hypothermic cardiac arrest patients resuscitated by CPB and concluded that of the 23 patients, 22 could be correctly classified as survivors or nonsurvivors based on the level of serum potassium and arterial pCO2 [7]. On the other hand, extreme parameters, including a core temperature of 13.7°C, a pH of 6.29 and a base excess of -36.5, have been reported in survivors [4]. This patient showed hypothermic cardiac arrest and asystole on arrival at our ED. At that time we could not identify whether he had undergone submersion or immersion. Arterial blood gas parameters on arrival (Table 1), namely a pH of 7.022, pCO2 of 46.0 mmHg, serum potassium of 5.6 mmol/l and base excess of -20.9 mmol/l, were comparatively good, compared to the above-mentioned prognostic values. Therefore, this patient might have been expected to resuscitate with a good prognosis.

**Table 1:** Laboratory data on admission

Biochemistry			Peripheral blood			Coagulation			Arterial blood gases		
T-bil	0.2	mg/dl	WBC	6,400	/μl	PT	14.4	s	pH	7.022	
TP	4.9	g/dl	RBC	395 × 10^4^	/μl	APTT	unmeasured		pCO_2_	46.0	mmHg
AST	43	IU/l	Hb	11.9	g/dl	Fib	194	mg/dl	pO_2 _9	2.3	mmHg
ALT	21	IU/l	Ht	38.5	%	AT-III	60	%	HCO_3_	11.4	mmol/l
LDH	194	IU/l	Plt	13.8 × 10^4^	/μl	FDP	< 5	μg/ml	B.E.	-20.9	mmol/l
AMY	104	IU/l							K	5.6	mmol/l
Na	155	mmol/l							Na	174	mmol/l
K	3.5	mmol/l							Cl	146	mmol/l
Cl	124	mmol/l							Lac	12.1	mmol/l
BUN	8	mg/dl									
CRE	0.6	mg/dl									
CPK	312	IU/l									
Glu	278	mg/dl									
CRP	< 0.1	mg/dl									

It is recommended that severe hypothermic patients be treated by active internal rewarming methods. These include an extracorporeal circulation device such as CPB, continuous renal replacement therapy (CRRT) and body cavity lavage [4]. CPB can rewarm patients the fastest and has the potential to support unstable hemodynamics, which may include the complex syndrome of rewarming shock.

In conclusion, this case represents successful recovery from severe hypothermic cardiac arrest with a good neurological outcome. For severe hypothermia, particularly in cardiac arrest patients, CPB is an extremely useful treatment device. The diagnostic criteria and management for the resuscitation of hypothermic cardiac arrest patients are still unclear, because we need to accumulate such cases.

## Consent

Written informed consent was obtained from the patient for publication of this case report and any accompanying images. A copy of the written consent is available for review by the Editor-in-Chief of this journal.

## Competing interests

The authors declare that they have no competing interests.

## Authors' contributions

KS drafted the manuscript. KT contributed advice for the manuscript. All authors read and approved the final manuscript.
